# Supplementary material for: Tead1a Initiates Transcriptional Priming Through the TEAD1a/YAP‐Notch1‐Spi1/Cebpα Axis to Promote Neutrophil Fate
Source: Adv Sci (Weinh). 2025 Aug 28;12(41):e05441. doi: 10.1002/advs.202505441 (PMC12591127; doi:10.1002/advs.202505441)
Supplement: Supplementary file 4 — Supplementary Dataset [file ADVS-12-e05441-s004.zip › 3-6.zip!/3-6/02-6.cluster/cluster16/Enrich/ego_BP.pdf]

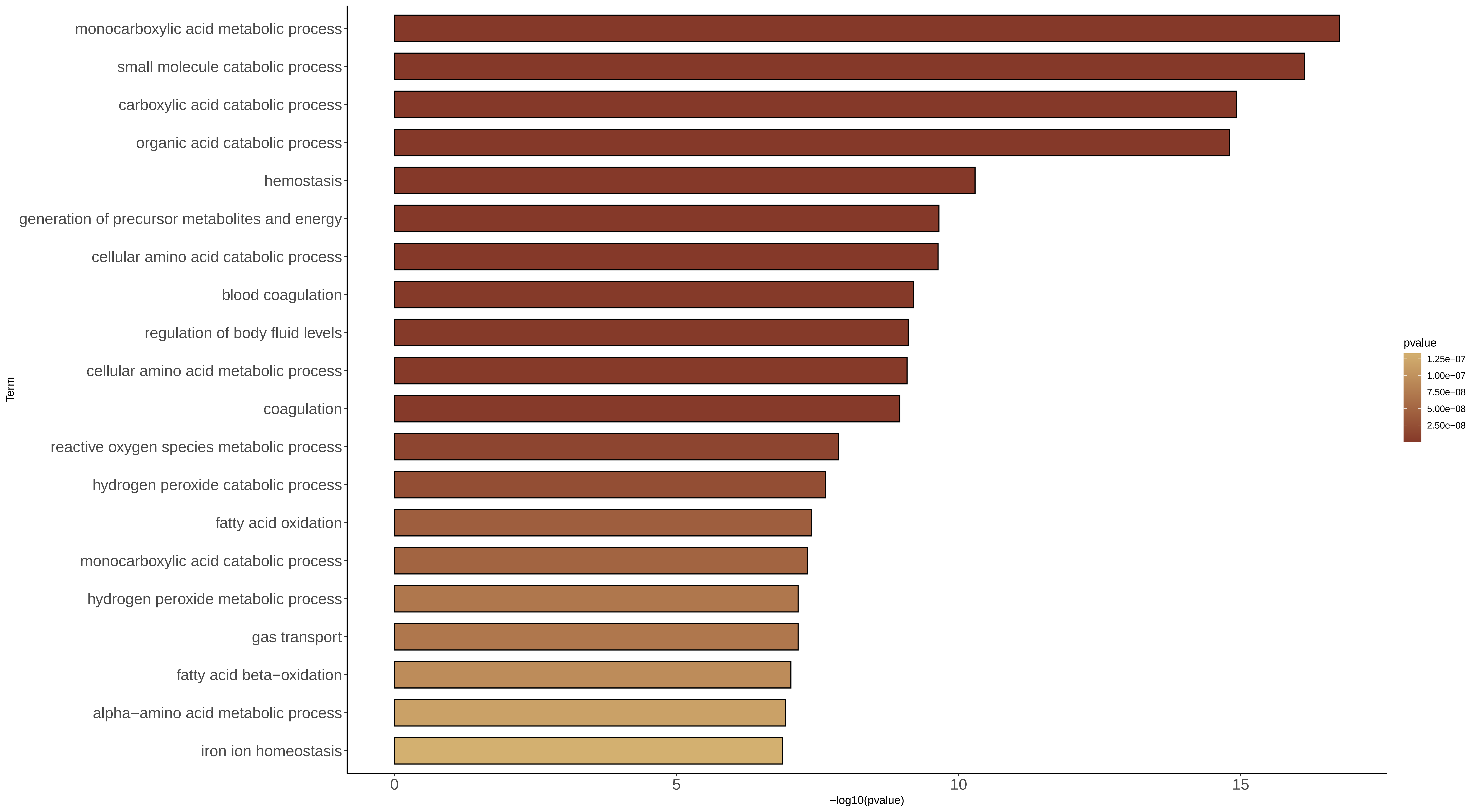

monocarboxylic acid metabolic process
small molecule catabolic process
carboxylic acid catabolic process
organic acid catabolic process
hemostasis
generation of precursor metabolites and energy
cellular amino acid catabolic process
blood coagulation
regulation of body fluid levels
cellular amino acid metabolic process
coagulation
reactive oxygen species metabolic process
hydrogen peroxide catabolic process
fatty acid oxidation
monocarboxylic acid catabolic process
hydrogen peroxide metabolic process
gas transport
fatty acid beta−oxidation
alpha−amino acid metabolic process
iron ion homeostasis
Term
0
5
10
15
−log10(pvalue)
pvalue
1.25e−07
1.00e−07
7.50e−08
5.00e−08
2.50e−08
